# ErbB3-binding protein EBP1 decreases ErbB2 levels via a transcriptional mechanism

**DOI:** 10.3892/or.2012.2186

**Published:** 2012-12-14

**Authors:** ARUNDHATI GHOSH, SMITA AWASTHI, ANNE W. HAMBURGER

**Affiliations:** 1 Greenebaum Cancer Center, University of Maryland, Baltimore, MD 21201, USA; 2 Department of Pathology, University of Maryland, Baltimore, MD 21201, USA

**Keywords:** EBP1, ErbB2, ErbB2 promoter

## Abstract

Ectopic expression of *EBP1*, an ErbB3-interacting protein, reduces the expression of the ErbB2 protein and mRNA. However, the mechanism of EBP1-induced decrease in ErbB2 mRNA levels has not yet been determined. Since EBP1 affects both transcriptional and post-transcriptional processes, we evaluated the ability of EBP1 to regulate ErbB2 transcription and RNA stability. We discovered that while wild-type EBP1 decreased the activity of a proximal ErbB2 promoter, EBP1 mutants unable to interact with the Sin3A transcriptional repressor inhibited activity to a lesser extent. EBP1 also decreased the activity of distal ErbB2 promoters. Chromatin immunoprecipitation analysis indicated that EBP1 bound both distal and proximal endogenous ErbB2 promoters in serum-starved conditions. The ErbB3 ligand heregulin (HRG) at growth-promoting concentrations reduced EBP1 binding to the ErbB2 promoter. Although endogenous EBP1 bound ErbB2 mRNA, *EBP1* overexpression or ablation of EBP1 protein by shRNA failed to alter ErbB2 mRNA stability. These results suggest that the major effect of EBP1 on ErbB2 mRNA levels is at the transcriptional level.

## Introduction

In our previous study, EBP1 was isolated as an ErbB3-binding protein (1). The ectopic expression of *EBP1* inhibits the growth of human breast cancer cells and induces cellular differentiation (2). The growth inhibiting properties of EBP1 are multi-faceted due to the fact that it binds DNA (3,4), RNA (5) and protein (6).

EBP1 is a member of the *PA2G4* family of DNA binding proteins, the prototype of which is a 42-kDa protein isolated from the fission yeast *Schizosaccharomyces pombe*(3). In addition, the murine homologue p38-2G4 was isolated as a DNA-binding protein from Ehrlich ascites cells (7). EBP1 binds to E2F1 consensus elements and represses the transcription of both *E2F1*(8) and the androgen receptor (AR)-regulated genes (9,10). EBP1 contains an autonomous C-terminal transcriptional repression domain that binds a transcriptional repression complex of HDAC2 and Sin3A (8,11).

In addition to its DNA-binding properties, EBP1 has been demonstrated as an RNA-binding protein. Squatrito *et al*(12) discovered that a pool of EBP1 localizes to the nucleolus, binds RNA and may be involved in ribosomal processing. Cytoplasmic EBP1 associates with the 40S subunit of mature ribosomes and has a role in protein translation. Sedimentation studies revealed that EBP1 copurified with eIF2α, a component of the translation initiation complex (13). EBP1 also affects mRNA stability. EBP1 binds to the 3′UTR of the AR mRNA and both destabilizes AR mRNA and represses AR mRNA translation (14). In contrast, EBP1 binds to the 3′UTR of bcl-2 mRNA and stabilizes β-globin-ARE bcl-2 transcripts (15). In addition, EBP1 binds HLA-DRA, HLA-DRB1 and HLA-DQA1 mRNA and regulates their respective mRNA levels (16).

The ErbB-3 ligand heregulin (HRG) regulates the phosphorylation, DNA and RNA binding activity of EBP1. EBP1 phosphorylation is increased at Ser363 (17) and Thr 261 (18) after HRG treatment. HRG enhances the ability of EBP1 to decrease the transcription of both E2F1 (4) and AR-regulated genes (19). In addition, EBP1 destabilizes AR mRNA in an HRG-inducible manner (14).

We previously reported that ectopic expression of *EBP1* decreases ErbB2 protein levels in human breast cancer cell lines (20,21). Ectopic expression of *EBP1* decreased steady-state levels of endogenous ErbB2 mRNA and decreased the activity of an ErbB2 proximal promoter reporter in cells which overexpress ErbB2 (21). The purpose of the present study was to further understand the basis of the ability of EBP1 to repress ErbB2 mRNA levels in ErbB2-overexpressing cells.

## Materials and methods

### Cell culture

The BT474 cell line was maintained at 37°C in a humidified atmosphere of 5% CO_2_ in air. Cell lines were routinely cultured in RPMI-1640 media supplemented with 10% FBS (Sigma, St. Louis, MO). The T47D EBP1-silenced and BT474 *EBP1*-overexpressing cell lines have been previously described (21).

### Plasmids

The ErbB2 promoter reporter plasmid encoding the -500 bp proximal ErbB2 promoter was a gift from Dr Chris Benz (University of California, San Francisco) (22). The -3798 and -6007 bp plasmids encoding distal promoters described by Delacroix *et al*(23) were a gift from Dr Jean Imbert (Université de la Méditerranée) (24). Wild-type and mutant *EBP1* expression plasmids used in this study were previously described (17).

### Chromatin immunoprecipitation (ChIP) assays

The method demonstrated by Shang *et al*(25) was used. Briefly, BT474 cells (3–4×10^6^) were grown in 150-mm dishes in RPMI-1640 medium supplemented with 10% FBS. After 24 h of culture, cells were transferred to serum-free RPMI-1640 media overnight. Cells were then left untreated or treated with 50 ng/ml of HRGβ1 (R&D Systems, Minneapolis, MN) for 20 min. Formaldehyde (1%) was added for 10 min and the reaction was quenched using 0.125 M glycine. Cells were harvested, pelleted and the pellets were resuspended in 0.3 ml of lysis buffer [1% SDS, 10 mM EDTA, 50 mM Tris-HCl (pH 8.1) and 0.5% NP-40] and 1X protease inhibitor cocktail (Roche, Indianapolis, IN). Chromatin was sheared to an average size of 500 bp by sonication using a Bioruptor ultrasonicator (Diagenode, Sparta, NJ) and diluted in ChIP dilution buffer (Millipore, Billerica, MA) to a final volume of 1 ml. A portion of the diluted cell supernatant (1%) was used to quantify the amount of DNA present in the samples. The EBP1 antibody (2 μg/reaction mixture) (Millipore) or negative ChIP validated pre-immune IgG (Sigma) as a control was added to the samples. ChIP validated agarose beads (Millipore) were added for another 6 h. Agarose-bead complexes were washed sequentially in low salt, high salt, LiCl and TE buffer provided in a kit from Millipore and extracted 2 times with freshly prepared elution buffer (1% SDS, 0.1 M NaHCO_3_). Eluates were pooled and incubated at 65°C for 6 h to reverse the formaldehyde cross-linking. DNA was purified by phenol-chloroform extraction and precipitated in the presence of 0.3 M sodium acetate and 1 μg glycogen in 2 volumes of ethanol at −20°C overnight. The DNA pellets were dissolved in 5 μl of sterile water. The gene-specific primer sequences were: -500 bp F, 5′-GGG GTC CTG GAA GCC ACA AGG TAA-3′ and R, 5′-ACT TTC CTG GGG AGC TTG CAT CCT-3′; -4600 bp F, 5′-TCC CCA GCA ACC TGT GCC TCA-3′ and R, 5′-ACC AGC CAG CTT GGG GTC AGA-3′; GAPDH F, 5′-ATG GTT GCC ACT GGG GAT CT-3′ and R, 5′-TGC CAA AGC CTA GGG GAA GA-3′. The MyiQ real-time PCR detection system and SYBR-Green PCR mix (Bio-Rad, Richmond, CA) were used to perform real-time PCR. The relative quantitation of targeted genes was determined by the comparative ΔΔCt (threshold) method using GAPDH as an internal control (14). Known quantities of input DNA were used to quantify the PCR products. In individual experiments, 3 wells were set up per data point. The data shown are the means ± SE from 3 independent experiments.

### Luciferase reporter assays

For the ErbB2 reporter assays, BT474 cells (1×10^5^) were plated in 6-well plates in complete media. When cells reached 50–60% confluency, they were transfected with 1 μg of an ErbB2 reporter plasmid and 5 ng of *Renilla (*pRL*)*-TK control plasmid using Lipofectamine 2000 (Invitrogen, Carlsbad, CA). Cell lysates were collected 48 h later and luciferase activity was assessed using a Promega Dual-Luciferase assay kit (Madison, WI). All transfection experiments were performed in triplicate wells and repeated 3 times. The activities of *Renilla* luciferase were used to normalize any variations in transfection efficiency. The data are expressed as relative light units (RLU) which is the ratio of ErbB2-luc RLU:pRL-TK RLU for each sample.

### Western blot analysis

Total cell extracts were prepared by direct lysis of cells with lysis buffer [50 mM Tris-HCl (pH 7.4), 1 mM EDTA, 250 mM NaCl, 1% Triton X-100, 0.5 mM DTT and 1X Complete™ protease inhibitor]. Protein concentrations were measured using a detergent compatible kit (Bio-Rad). Proteins (30 μg/well) were resolved by SDS-PAGE, transferred to PVDF membranes and immunoblotted as previously described (2). The EBP1 antibody was obtained from Millipore and the anti-actin FLAG and GAPDH antibodies were from Sigma.

### RNP immunoprecipitation assays

For immunoprecipitation (IP) of endogenous ErbB2 mRNA-EBP1 protein complexes (RNP), cell lysates (1.5 mg) were incubated for 2 h at 4°C with protein A-Sepharose beads (Calbiochem) that had been precoated with 3 μg of pre-immune IgG (Sigma) or antibodies recognizing EBP1 or HuR (Santa Cruz Biotechnology, Santa Cruz, CA). Beads were washed with NT2 buffer [50 mM Tris-HCl (pH 7.4), 150 mM NaCl, 1 mM MgCl_2_ and 0.05% NP-40], incubated with 20 units of RNase-free DNase I (15 min, 30°C), followed by incubation with 100 μl NT2 buffer containing 0.1% SDS and 0.5 mg/ml proteinase K for another 30 min at 55°C. The RNA isolated from the IP was converted to cDNA using gene-specific primer pairs F, 5′-GGGAAGAATGGGGTCGTCAAA-3′ and R, 5′-CTCCTCCCTGGGGTGTCAAG-3′ and amplified by real-time quantitative PCR as described.

### Analysis of mRNA stability

Cells were serum-starved overnight and then treated with actinomycin D (5 μg/ml). Cells were harvested at sequential time points following actinomycin addition. Total RNA was extracted with Trizol and DNase treated for RT-qPCR analysis. The data represent the means ± SE of three to five independent experiments.

### Statistical analysis

Results were analyzed using a two-tailed Student’s t-test. A value of P<0.05 was considered to indicate a statistically significant difference.

## Results

### EBP1 affects ErbB2 promoter activity

**Figure 1:**
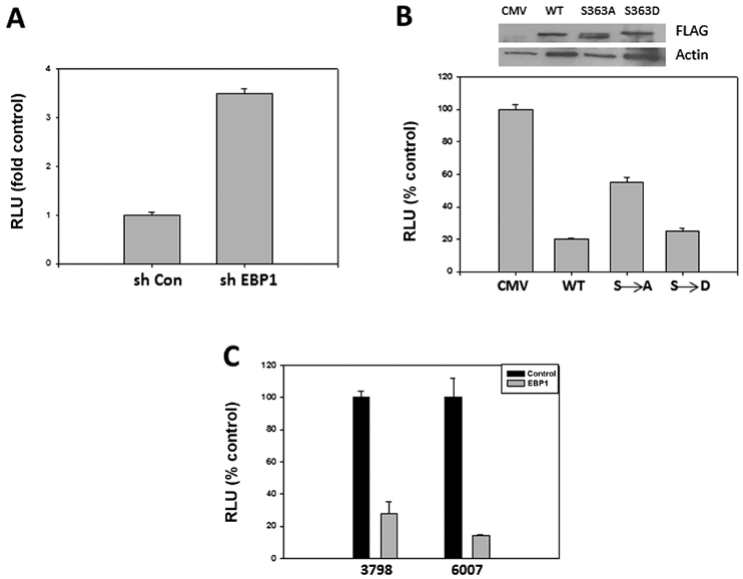
Effect of EBP1 on ErbB2 promoter activity. (A) Silencing of EBP1 increases ErbB2 promoter activity. T47D cells in which EBP1 was silenced by shRNA were transiently transfected with the ErbB2 proximal promoter luciferase construct and pRL-TK. After 48 h, cells were lysed and the relative luciferase units were determined as described in Material and methods. Each bar represents the mean ± SD of 8 wells. The graph is representative of 2 independent experiments. (B) Transcriptional repression by EBP1 is partially dependent on S363 phosphorylation. BT474 cells were transiently transfected with a CMV10 control plasmid, wild-type EBP1, the S363A or S363D EBP1 mutant, ErbB2-luc (proximal promoter) and pRL-TK. Luciferase activity was determined as described in A. Expression levels of the FLAG-tagged EBP1 constructs are shown. (C) EBP1 inhibits the activity of distal promoters. BT474 cells were transiently transfected with the ErbB2 distal (-3798) or (-6007) promoter luciferase constructs, CMV10-EBP1 and pRL-TK. After 48 h, cells were lysed and relative luciferase units were determined as described in A.

We previously showed that ectopic expression of *EBP1* decreased the activity of an *ErbB2* luciferase reporter that encodes the 500-bp proximal promoter upstream from the transcription start site (21). We, therefore, aimed to determine the effect of the inhibition of EBP1 expression on ErbB2 promoter activity. T47D cells were used in which EBP1 expression was silenced by shRNA (Fig. 3C) and ErbB2 expression was increased (21). The activity of the ErbB2 promoter construct was increased 3-fold as compared to the shRNA controls (Fig. 1A).

To further explore the mechanism of the EBP1-induced transcriptional repression, we assessed the ability of EBP1 mutants to inhibit ErbB2 promoter activity. EBP1 phosphorylation at Ser363 is required for EBP1 to bind Sin3A and inhibit the transcription of E2F1-regulated promoters (17). We examined the ability of the non-phosphorylatable S363A and phosphomimetic S363D mutants to affect ErbB2 promoter activity. Previously published data indicated that the subcellular distribution of the mutants was similar to that of wild-type EBP1 (17). The expression of wild-type and the EBP1 mutants was approximately the same (Fig. 1B, upper image). BT474 cells were transfected with pRL-TK, ErbB2-Luc and either CMV10, CMV10-EBP1, CMV10-EBP1 S363A or EBP1 S363D. CMV10-EBP1 significantly repressed ErbB2 promoter activity by 79% as compared to the vector control (P<0.001). The S363A mutation significantly reduced EBP1-mediated transcriptional repression (P<0.05). The activity of the S363D mutant was not significantly different than that of the wild-type (Fig. 1B).

Distal ErbB2 promoters are important in regulating ErbB2 levels in ErbB2-overexpressing cells (23). The ability of EBP1 to affect the activity of reporter vectors encoding -6007 and -3798 sites upsteam of the transcriptional start site were assessed. We discovered that EBP1 inhibited the activity of the -3798 promoter by 71% and the -6007 by 90% (Fig. 1C).

### EBP1 interacts with the distal and proximal endogenous ErbB2 promoters

**Figure 2:**
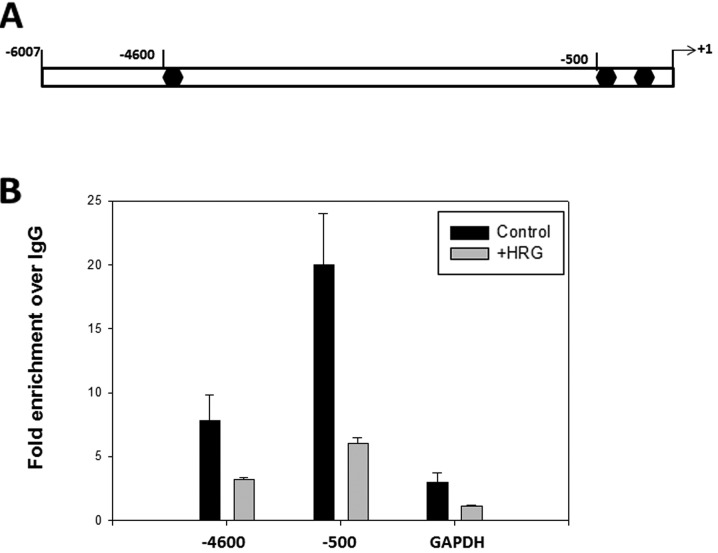
EBP1 is recruited to ErbB2 5′ flanking sequences in BT474 cells. (A) Schematic representation of the 5′ region of the ERBB2 gene. ^+^1 indicates the major transcription start site. Hexagons indicate AP2 sites. (B) BT474 cells were serum-starved overnight and then treated with HRG β1 (50 ng/ml) for 20 min. Quantitative ChIP assays were performed using pre-immune IgG or EBP1 antibody as described in Materials and methods. Means ± SE of 3 independent experiments.

We used ChIP assays to determine whether EBP1 may assemble on endogenous proximal and distal ErbB2 promoters. Although the proximal ErbB2 promoter is active in cell lines expressing both high and low levels of ErbB2, distal promoter sites are important in cells in which ErbB2 is overexpressed (23) (Fig. 2A). We were additionally interested in the ability of the ErbB3 ligand HRG to affect EBP1 binding. BT474 cells were serum-starved and then treated with HRG for 20 min. EBP1 associated with both the -4600 and -500 bp elements previously demonstrated to be implicated in ErbB2 transcriptional regulation (23). The distal -4600 bp site was immunoprecipitated to a lesser extent compared to the 500 bp site. In contrast, the association of EBP1 with the GAPDH promoter was not significantly different compared to the IgG control indicating that the association of EBP1 with the ErbB2 promoter was specific. HRG treatment decreased the binding of EBP1 to both recognition sites (Fig. 2).

### EBP1 does not influence ErbB2 mRNA decay

**Figure 3:**
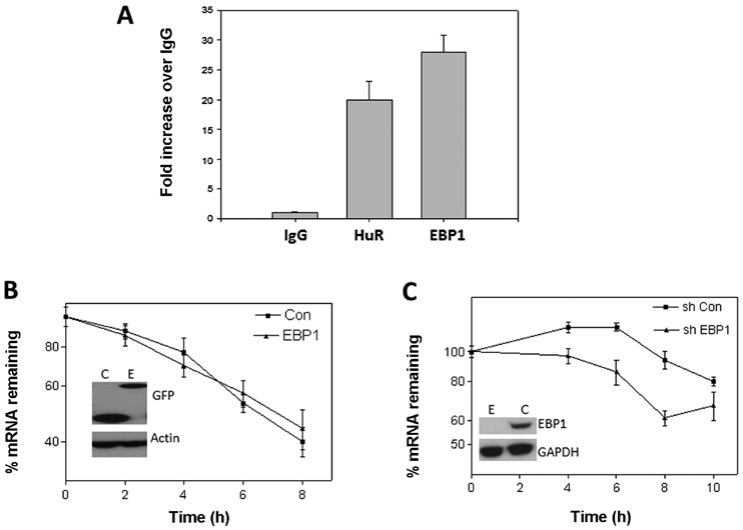
Effect of EBP1 on ErbB2 mRNA decay. (A) Binding of endogenous HuR and EBP1 to endogenous ErbB2 mRNA. BT474 lysates were immunoprecipitated with antibody to HuR, EBP1 or the control IgG. Total RNA was isolated using Trizol, and qRT-PCR using ErbB2-specific primers was used to determine whether HuR and EBP1 were associated with ErbB2 mRNA. All data are expressed as the fold increase over IgG. (B) Effect of ectopic expression of EBP1 on ErbB2 mRNA stability. The stability of ErbB2 and GAPDH mRNA was analyzed in BT474 cells transfected with the vector control (EGFP) or with EGFP-EBP1. Total cellular RNA was isolated at the indicated times after treatment with actinomycin D. The remaining levels of ErbB2 and GAPDH mRNAs were measured by RT-qPCR analysis. Values are the means ± SE of triplicates. Results are representative of 3 independent experiments. Inset image = western blot analysis of GFP-tagged proteins. (C) Effect of silencing of EBP1 expression on ErbB2 mRNA stability. The stability of ErbB2 and GAPDH mRNA was analyzed in shRNA control (C) and shEBP1 transduced (E) T47D cells. Total cellular RNA was isolated at the indicated times after treatment with actinomycin D. The remaining levels of ErbB2 and GAPDH mRNAs were measured by RT-qPCR analysis. Results depicted are the averages of 5 independent experiments ± SE. Inset image = expression levels of EBP1 and GAPDH.

The decreased steady-state levels of ErbB2 mRNA observed after EBP1 overexpression may have resulted from either decreased transcription or decreased mRNA stability. A previous study demonstrated that endogenous EBP1 binds AR mRNA and that ectopic expression of *EBP1* decreases the levels of AR mRNA by destabilizing AR mRNA in prostate cancer cell lines (14). We first examined the association of endogenous EBP1 with endogenous ErbB2 mRNA by RNA-IP analysis. ErbB2 mRNA was detected in the EBP1 immunoprecipitates by reverse transcription using primers for the ErbB2 coding region. As reported, HuR also bound ErbB2 mRNA (22) (Fig. 3A). Actin mRNA was not enriched in EBP1 immunoprecipitates compared to the control IgG (data not shown). To examine if EBP1 is involved in the post-transcriptional regulation of ErbB2, we measured the half-life of ErbB2 mRNA in control and BT474 cells expressing *GFP-EBP1* in actinomycin D pulse chase experiments. ErbB2 mRNA stability was not altered by the overexpression of *EBP1* (Fig. 3B). Similarly, ablation of EBP1 in T47D cells did not significantly affect overall ErbB2 mRNA stability (Fig. 3C).

## Discussion

ErbB heterodimers and their interacting partners are important in breast cancer development (26). We previously showed that the ErbB3-binding protein EBP1 inhibited the growth of ErbB2/ErbB3-expressing breast cancer cell lines partially by downregulating the protein levels of ErbB2 (20). Ectopic expression of *EBP1* resulted in decreased steady-state levels of ErbB2 mRNA (21). However, we did not assess whether the decrease in steady-state mRNA levels was due to changes in ErbB2 transcription or mRNA stability. In this current study the EBP1-induced changes in ErbB2 mRNA were possibly due to the decreased transcription of the *ERBB2* gene.

We first examined the effects of silencing of endogenous EBP1 on the activity of an ErbB2 proximal promoter. We discovered that the activity of the ErbB2 proximal promoter was enhanced by silencing EBP1 expression. This coincides with previous data indicating that the ectopic expression of *EBP1* reduced promoter activity in ErbB2-overexpressing cells (21). To determine whether the effects of EBP1 were mediated by its ability to bind DNA, we examined the activity of a non-DNA-binding mutant. EBP1 was shown to bind DNA through it interactions with the transcriptional repressor Sin3A (11). Phosphorylation of Ser363 is required for this interaction (17). We demonstrated that the *EBP1* S363A non-phosphorylatable mutant had a decreased ability to inhibit reporter activity. These findings suggest that the interaction of EBP1 with Sin3A may affect ErbB promoter activity.

To further confirm the role of endogenous EBP1 in ErbB2 transcription, we determined whether EBP1 was recruited to the ErbB2 promoter. Although a -500 bp promoter has been shown to be active in all breast cancer cell lines tested (27,28), a promoter fragment between -6007 and -3798 actively enhances transcription in ErbB2-overexpressing cells (23). The transcription factor AP-2, known to be important for *ERBB2* transcription, strongly binds the -500 bp region at multiple sites and the -4600 bp region to a lesser extent at one site. We similarly found that EBP1 bound both proximal and distal ErbB2 promoters and that the binding of EBP1 to the distal site was much less than that found at the proximal binding site. EBP1 does not bind DNA directly (4) and it is possible that it may be interacting with AP-2 at both these sites in the ErbB2 promoter. EBP1 may also interact with other proteins such as Ets family members (27), Wwox (29) and GATA-4 (24) that bind and regulate the activity of the ErbB2 promoter. Currently, studies are underway in our laboratory to examine EBP1 interactions with other relevant transcriptional factors. Finally, treatment with the ErbB2/3 ligand HRG resulted in decreased EBP1 occupancy at both the ErbB2 promoters. This finding contradicts our previous research demonstrating that HRG increased the binding of EBP1 to AR-regulated promoters (11). The reasons for this discrepancy are unclear. However, HRG inhibits the growth of LNCaP cells, while it stimulates the growth of BT474 cells (30,31). In our study, 50 ng/ml of HRG β1 stimulated cell growth ~30% (data not shown) similar to previous reports. Thus, the decreased occupancy of EBP1, which is a transcriptional repressor, may be associated with increased proliferation.

We discovered that although EBP1 binds ErbB2 mRNA, neither ectopic expression of *EBP1* nor abrogation of EBP1 protein expression affected ErbB2 mRNA stability. This finding is in contrast to our previous work indicating that the overexpression of *EBP1* destabilizes AR mRNA (14). The inability of changes in EBP1 expression to affect ErbB2 mRNA stability remains unclear. However, Scott *et al*(22) discovered that the RNA binding protein HuR binds to the 3′ UTR of ErbB2 transcripts and destabilizes ErbB2 via a Class II HDAC-6 mediated mechanism. It is possible that HDAC-6 is required for ErbB2 mRNA destabilization. Although EBP1 has been demonstrated to bind nuclear class I HDAC2, it does not bind class II HDACs (8). Thus, the failure to recruit HDAC6 may be responsible for the inability of EBP1 to destabilize ErbB2 mRNA.

In summary, we found that the ectopic expression of *EBP1* suppresses ErbB2 levels via a transcriptional mechanism. This study suggests that the mode by which EBP1 regulates protein levels may depend on the target gene and cellular context. An understanding of the factors that regulate the ability of EBP1 to affect transcriptional and/or post-transcriptional mechanisms may clarify the manner in which one protein regulates a variety of downstream pathways.
